# Construction and verification of a nomogram to predict the probability of venous thromboembolism in lung cancer patients

**DOI:** 10.3389/fmolb.2026.1671697

**Published:** 2026-09-04

**Authors:** Rong Deng, Jing Wu, Fei Tang, Yan Li, Qin Zhong, Ying Tong, Tingting Ni, Yu Zhang

**Affiliations:** 1 Oncology Department, Guizhou Provincial People’s Hospital, Guiyang, China; 2 Department of Pathology, Guizhou Provincial People’s Hospital, Guiyang, China; 3 NHC Key Laboratory of Pulmonary Immune-related Diseases, Guiyang, Guizhou, China

**Keywords:** decision making, lung cancer, nomogram, predictive factors, venous thromboembolism

## Abstract

**Background:**

Lung cancer (LC) patients account for 20% of all cancer-related venous thromboembolism (VTE) events, which is the second leading cause of mortality in these patients. However, there are no LC-specific risk scores for VTE prediction. Therefore, we considered that an LC-specific nomogram would more accurately predict VTE probability for these patients than the current widely used VTE risk scores.

**Methods:**

A total of 676 patients from the Guizhou Provincial People’s Hospital (January 2016 to September 2021) were included in this study, of which 169 LC patients who developed VTE were time-matched with 507 (1:3 ratio) LC patients without VTE. These patients were randomly divided at a 2:1 ratio to form primary (451) and validation (225) cohorts. The accuracy of six VTE risk scores was assessed by producing area under the receiver operating characteristic (ROC) curves (AUC). A multivariate analysis was employed to select predictive features, which were then used to construct a nomogram for VTE prediction.

**Results:**

Among the scoring methods, COMPASS-CAT had the highest AUC (0.799). The multivariate analyses revealed that acute infection, bed rest (>3 days), D-dimer level >1.47 μg/mL, adenocarcinoma, and carcinoembryonic antigen (CEA) >10.935 ng/mL were independent predictors of VTE risk for LC patients. The nomogram constructed using these factors enabled VTE prediction with a concordance index of 0.882 and an AUC of 0.894.

**Conclusion:**

This article describes the construction and validation of a new nomogram for VTE prediction in LC patients, which has a predictive performance that is higher than any of the widely used conventional risk assessment tools.

## 1 Introduction

Lung cancer (LC) is not only the most common malignancy worldwide but is also the leading cause of cancer-associated mortality (Ferlay et al., 2019). Malignant tumors are among those associated with venous thromboembolism (VTE), which includes deep vein thrombosis (DVT) and pulmonary embolism (PE). LC patients have a 7%–13% reported incidence of VTE, which is a 13-fold greater risk than the general population (Cushman, 2007; Petterson et al., 2015). The fact that LC-associated VTE ranks highest in terms of absolute number among VTEs can be attributed to the leading incidence of LC itself. Cancer-associated VTE has been associated with substantial inpatient length of stay, delays in cancer treatment, and mortality. VTE is the second leading cause of death in LC patients (Khorana et al., 2007; Howlett et al., 2020). Because VTE is a preventable condition, the cornerstone of its clinical management relates to the early identification of high-risk groups. Existing evidence suggests that effective risk assessment and prevention can reduce the absolute VTE risk in cancer patients by 50% (Di Nisio et al., 2012).

The Caprini (Hachey et al., 2016) (applicable to surgical and cancer patients), Padua (Barbar et al., 2010) (hospitalized patients), Wells (Wells et al., 1997) Geneva (Nendaz et al., 2014) (for PE), Khorana (Khorana et al., 2008), and COMPASS-CAT (Gerotziafas et al., 2017) (cancer patients) scores are most widely used methods for VTE risk scoring. Among these, only the Khorana and COMPASS-CAT scores are tumor-related VTE risk score methodologies. The Khorana score is the most widely used risk assessment tool for cancer patients (Khorana et al., 2008). However, many studies have found Khorana scoring to perform poorly for LC-associated VTE risk prediction (Mulder et al., 2019; Xiong et al., 2021; Overvad et al., 2022). A meta-analysis of the Khorana scores from 34,555 cancer patients (55 cohorts) determined that the risk of developing LC-associated VTE within the first 6 months was 6.4% in the high-risk group (van Es et al., 2020). Mansfield et al. (2016) collected data from 719 LC patients and reached similar conclusions using Khorana scoring, finding cumulative VTE incidence of 5.2%, 6.2%, 7.2%, and 10.3% at 3 months, 6 months, 12 months, and 24 months, respectively. The Khorana score has also been associated with a low sensitivity of 10%–25% for LC (Rupa-Matysek et al., 2018; Alexander et al., 2019). Rupa-Matysek et al. (2018) found that the COMPASS-CAT score was the most accurate method for assessing the risk of VTE occurrence in LC patients; however, the sample size (n = 118) was relatively small. Additionally, the specificity was decreased compared to the Khorana score, to 35%–51%, meaning approximately two-thirds to half of the patients that did not develop VTE were classified as high risk (Rupa-Matysek et al., 2018; Syrigos et al., 2018).

Moreover, these tools are not specifically designed for lung cancer. Hence, we thought that an LC-specific risk assessment tool would outperform the six tools that are currently widely used to generate VTE risk scores for LC patients. In recent years, nomograms have emerged as reliable and alternative tools for prognostic and predictive modeling, integrating pathological, molecular, and clinical characteristics of diseases to establish an evaluation system for predicting individual patient outcomes that has been widely validated across multiple cancer types (Zuo et al., 2021; Feng et al., 2022; Zuo et al., 2022; Zuo et al., 2023). This assessment nomogram was constructed to integrate LC characteristics and laboratory values to help clinicians accurately predict VTE and improve clinical decision making to better apply thrombus prophylaxis in high-risk LC patients.

## 2 Methods

### 2.1 Patients

This study assessed hospitalized LC patients who developed VTE (DVT or PE) from January 2016 to September 2021 at the Guizhou Provincial People’s Hospital (CN).

Sample size calculation: We calculated the sample size based on the minimum number of events per variable (EPV) criterion, which is widely recommended for developing clinical prediction models to ensure model stability and avoid overfitting. The standard formulas are:

$${\text{E}}_{\text{min}}=p\times 10\;\left(\text{Minimum number of events}\right)$$

$$N=\text{Emin}/\pi \;\left(\text{Total sample size}\right)$$

$${\text{N}}_{\text{final}}=N\times \left(1+r\right)\;\left(\text{Final sample size with allowance}\right)$$

Where:

p:Number of independent variables in the final model

$${\text{E}}_{\text{min}}:\text{Minimum required outcome events}$$

π:Expected event rate

$$r:\text{Allowance for loss to follow}‐\text{up }\left(10\%\right)$$

Based on a comprehensive review of six widely used VTE risk scores (Caprini: 33 variables, Padua: 11 variables, Wells: 7/8 variables, Geneva: 8 variables, Khorana: 5 variables, COMPASS-CAT: 8 variables), we selected the median value and set 8 as the number of predictor variables to be included (p = 8). According to the literature, the incidence of VTE in patients with lung cancer ranges from 7% to 13%. Therefore, we used 13% for the calculation, that is, π = 13%. With an EPV of 10 and 8 covariates, the recommended minimum number of events = 10 × 8 = 80, requiring a total sample size of 80/0.13 ≈ 615. After adding a 10% buffer for potential loss to follow-up, the target sample size was determined to be 676 patients.

We identified 169 patients with LC who developed VTE and 507 patients (1:3) with only LC who were entered into a time-matched retrospective quality assurance analysis. Inclusion criteria were as follows: (1) a histological LC diagnosis; (2) patients aged ≥18 years; (3) DVT events that were confirmed via venous ultrasound or computed tomography venous angiogram, or PE diagnosed via computed tomography (CT) or CT pulmonary angiogram. Patients with multiple tumors, age <18 years, insufficient information, or unavailability for follow-up were excluded. This study was approved by the Guizhou Provincial People’s Hospital ethics committee and was conducted in accordance with the Declaration of Helsinki.

### 2.2 Data collection

The patient data were collected from the hospital electronic medical records system, including VTE- and LC-related clinicopathological characteristics. The data also included: basic patient information (age, gender, co-occurring diseases and smoking history); pathological information (pathological type, differentiation and pathological diameter), genetic test results (with or without genetic mutation, mutant gene type, and mosaic/heterogeneity status), TNM clinical staging system information, laboratory examination results (routine blood tests, coagulation function and tumor markers), and treatment information.

Caprini, Padua, Wells, Geneva, Khorana, and COMPASS-CAT scores were evaluated by two independent researchers, and any discrepancies were resolved by group discussion until a consensus was reached. Patients with high VTE risk assessed by all the scoring methodologies were defined as VTE likely, whereas those with non-high risk of VTE were defined as VTE unlikely.

### 2.3 Diagnosis and classification of VTE

All VTE events, including DVT and PE, were objectively diagnosed. DVT was defined as the formation of a blood clot in a deep vein, most commonly the legs. PE is a result of detachment of a clot that travels to the pulmonary artery. The DVT events were confirmed via venous ultrasound or a computed tomography venous angiogram, while PE was diagnosed via computed tomography (CT) or CT pulmonary angiogram.

### 2.4 Statistical analysis

The statistical analyses were conducted using IBM SPSS 26.0 software. Continuous variables are presented as means plus/minus (±) the standard deviation or medians. Categorical variables are described using frequencies (percentages). The sensitivity, specificity, positive predictive value (PPV), negative predictive value (NPV), false positive rate (FPR), false negative rate (FNR), positive likelihood ratio (PLR), negative likelihood ratio (NLR), diagnostic odds ratio (DOR), and Youden index (YI) were used for the assessment of VTE development, and compared among the Caprini, Padua, Wells, Geneva, Khorana, and COMPASS-CAT scores. The area under the receiver operating characteristic (ROC) curves (AUC) were used to compare the accuracy of each VTE risk score system. A univariate analysis was performed to illustrate the preoperative risk factors for VTE. Factors with a significant *P* value (*P*=<0.05) in the univariate analysis were further analyzed using a multivariable logistic regression analysis, for which the odds ratios (ORs) and 95% confidence intervals (CIs) were calculated. The optimal cutoff values for platelet (PLT), white blood cell count (WBC), D-dimer (DD), and fibrinogen (Fbg), gastrin-releasing peptide precursor (ProGRP), carcinoembryonic antigen (CEA), neuron-specific enolase (NSE), cytokeratin 19 fragment (CYFRA21-1), and squamous cell carcinoma antigen (SCC) were chosen based upon the ROC curve, and the YI was obtained using the sensitivity and specificity for each point. Furthermore, in our study, patients with incomplete data were regarded as meeting the exclusion criteria and were therefore not included in the final analysis. As a result, no missing data were present in our final study cohort, and all included patients had complete information for all variables. *P*=<0.05 was used as the threshold when considering statistical significance.

The nomogram model was constructed based upon a multivariate analysis through the “R” package. The discrimination performance of the nomogram was quantitatively assessed using the concordance index (C-index). Actual and predicted incidence rates were compared using calibration plots, and calibration was internally validated using 1000 bootstrap resample repetitions. The clinical value of the model was assessed using decision curve analysis (DCA), which was plotted with the threshold probability and the net benefit.

## 3 Results

### 3.1 Clinical characteristics

A total of 676 patients were enrolled in this study. Within that cohort, 169 patients with LC who developed VTE were identified and time-matched in a 1:3 ratio with 507 patients with LC alone. The VTE group included 169 patients, of which 92 (54.4%) had PE, 49 (29.0%) had DVT, and 28 (16.6%) had both PE and DVT. There were 464 males and 212 females, with a median age of 61 years (23–98 years). In terms of cancer stage, 100 patients (14.8%) had early stage (I and II) LC, whereas 576 (85.2%) patients had progressed to late stage (III and IV) disease. The main pathological types were adenocarcinoma (454 cases, 67.2%), squamous cell carcinoma (128 cases, 18.9%), and small cell lung cancer (85 cases, 12.6%); nine patients (1.3%) had LC of other pathological types. Half of the patients (344 patients; 50.9%) received chemotherapy, 67 patients (9.9%) received radiotherapy, 82 patients (12.1%) received targeted therapy, 59 patients (8.7%) received antiangiogenic therapy, 15 patients (2.2%) received immunotherapy, and 332 patients (49.1%) did not receive any treatment.

In order to better validate the model, we randomly divided the overall enrolled population into the primary cohort (451) and validation cohort (225) at a ratio of 2:1 using computer-generated random numbers. There were no significant differences between the primary and validation cohorts (Table 1).

**TABLE 1:** Lung cancer patient clinicopathological characteristics in the primary and validation cohorts.

Characteristics	Primary cohort, n = 451	Validation cohort, n = 225	χ^2^ value	*P*
Age			0.089	0.765
≤60 years	213	109		
>60 years	238	116		
Sex			1.283	0.257
Male	316	148		
Female	135	77		
Body mass index (BMI) (kg/m^2^)			0.004	0.951
≥25	69	35		
<25	266	133		
Unknown	116	57		
Smoking status			1.373	0.241
Yes	252	115		
No	199	110		
Hypertension			0.055	0.815
Yes	116	56		
No	335	169		
Diabetes			0.701	0.403
Yes	56	23		
No	395	202		
Coronary heart disease			1.430	0.232
Yes	27	19		
No	424	206		
Acute myocardial infarction			1.711	0.191
Yes	2	4		
No	449	221		
Cardiac failure			0.379	0.538
Yes	1	2		
No	450	223		
Cerebral ischemic stroke			2.399	0.121
Yes	5	7		
No	446	218		
Respiratory failure			0.040	0.841
Yes	34	16		
No	417	209		
Acute infection			2.702	0.100
Yes	93	59		
No	358	166		
Rheumatic disease			0.379	0.538
Yes	1	2		
No	450	223		
Bed rest (>3 days)			<0.001	0.984
Yes	154	77		
No	297	148		
Lower limb edema			2.458	0.117
Yes	24	19		
No	427	206		
Transfusion			2.593	0.107
Yes	10	10		
No	441	215		
Central venous catheter			0.414	0.520
Yes	30	18		
No	421	207		
VTE history			0.022	0.882
Yes	15	7		
No	436	218		
Pathology			1.482	0.686
Small-cell lung cancer (SCLC)	53	32		
Adenocarcinoma	302	152		
Squamous carcinoma	90	38		
Other	6	3		
Differentiation			0.948	0.330
High/Moderately	21	8		
Poorly	27	17		
Unknown	403	200		
T-stage			2.108	0.550
T1	95	41		
T2	138	62		
T3	55	31		
T4	163	91		
N-stage			1.018	0.797
N0	99	51		
N1	21	14		
N2	159	80		
N3	172	80		
M-stage			0.065	0.798
M0	181	88		
M1	270	137		
Clinical stage			0.200	0.978
Stage I	30	15		
Stage II	36	19		
Stage III	115	54		
Stage IV	270	137		
Gene mutation			0.351	0.554
Mutant type	106	49		
EGFR+	78	43		
ALK+	17	4		
ROS1+	6	1		
Others	5	1		
Wild type	57	22		
Unknown	288	54		
White blood cell count >8.605 × 10^9^/L			1.048	0.306
Yes	154	68		
No	297	157		
Platelet count >349.5 × 10^9^/L			0.023	0.880
Yes	60	29		
No	391	196		
D-dimer level >1.47 μg/mL			2.275	0.131
Yes	241	134		
No	210	91		
Fbg >4.895 g/L			0.143	0.705
Yes	102	48		
No	349	177		
CEA >10.935 ng/mL			2.559	0.110
Yes	133	80		
No	318	145		
NSE >12.845 μg/L			0.927	0.336
Yes	247	132		
No	204	93		
CY211 > 9.765 μg/L			2.664	0.103
Yes	198	84		
No	253	141		
proGRP > 38.165 ρg/mL			1.745	0.186
Yes	357	168		
No	94	57		
SCC > 0.33 ng/mL			0.493	0.483
Yes	432	218		
No	19	7		
Surgery			1.918	0.166
Yes	53	35		
No	398	190		
Chemotherapy			0.807	0.4369
Only chemotherapy	142	62		
Chemotherapy combined with other treatments^a^	91	49		
Untreated	218	114		
Targeted therapy			0.021	0.884
Yes	55	27		
No	178	84		
Untreated	218	114		
Antiangiogenic therapy			0.389	0.533
Yes	42	17		
No	191	94		
Untreated	218	114		
Immunotherapy			0.429	0.512
Yes	9	6		
No	224	105		
Untreated	218	114		
Radiotherapy			2.455	0.117
Yes	40	27		
No	193	84		
Untreated	218	114		

VTE, venous thromboembolism; BMI: body mass index; SCLC, small cell lung cancer; EGFR, epidermal growth factor receptor; ALK, anaplastic lymphoma kinase; ROS-1, ROS proto-oncogene 1; Fbg, fibrinogen; CEA, carcinoembryonic antigen; NSE, neuron-specific enolase; CY21-1, cytokeratin 19 fragment; ProGRP, gastrin-releasing peptide precursor; SCC, squamous cell carcinoma antigen.

a Other treatment including radiotherapy, antiangiogenic therapy, and immunotherapy.

### 3.2 VTE development score accuracy assessment

**FIGURE 1:**
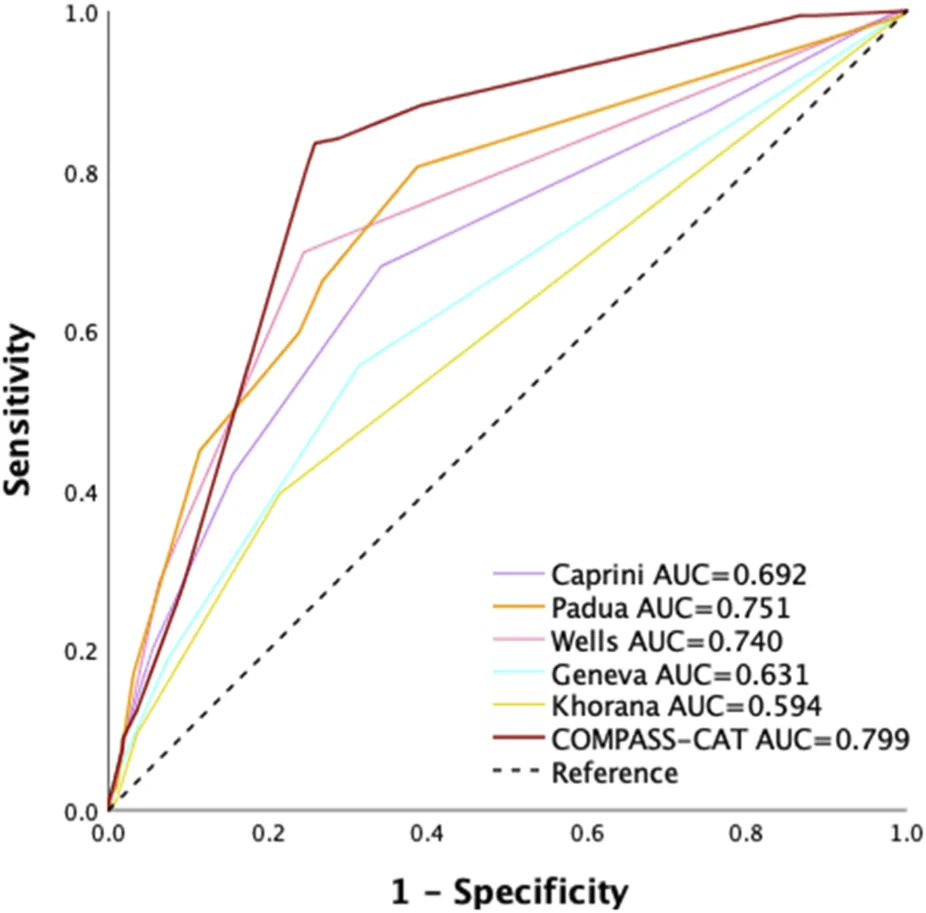
ROC curves for the Khorana, Caprini, Padua, Wells, Geneva, and COMPASS-CAT scores. ROC: receiver operating characteristic.

The sensitivity, specificity, PPV, NPV, FPR, FNR, PLR, NLR, DOR, and YI that were generated for all the VTE assessment scores are presented in Table 2. The sensitivity of the Caprini, Padua, Wells, Geneva, Khorana, and COMPASS-CAT scores was 68.0%, 80.5%, 69.8%, 55.6%, 9.5%, and 79.9%, respectively; whilst the specificity of these methods was 65.9%, 61.5%, 75.5%, 61.5%, 92.5%, and 75.3%, respectively. The COMPASS-CAT score had the highest AUC (0.799), followed by the Padua (0.751), Wells (0.740), Caprini (0.692), Geneva (0.631), and Khorana scores (0.594) (Figure 1).

**TABLE 2:** Accuracy assessments for the VTE development scores.

Variables	Caprini	Padua	Wells	Geneva	Khorana	COMPASS-CAT
Sensitivity-%	68.0	80.5	69.8	55.6	9.5	79.9
Specificity-%	65.9	61.5	75.5	61.5	92.5	75.3
PPV-%	39.9	41.1	48.8	41.1	29.6	51.9
NPV-%	86.1	90.4	88.2	90.4	75.4	91.8
FPR-%	34.1	38.5	24.5	38.5	7.5	48.1
FNR-%	32.0	19.5	30.2	44.4	90.5	8.2
PLR	1.99	2.1	2.9	1.44	1.3	3.24
NLR	0.49	0.3	0.4	0.72	0.98	0.27
DOR	4.06	6.6	7.2	3.07	1.3	12.00
YI	0.34	0.42	0.45	0.17	0.02	0.55

VTE, venous thromboembolism; PPV: positive predictive value, NPV: negative predictive value, FPR: false positive rate, FNR: false negative rate, PLR: positive likelihood ratio, NLR: negative likelihood ratio, DOR: diagnostic odds ratio, YI: Youden index.

### 3.3 Risk factors for VTE

In both cohorts, the univariate analysis revealed that respiratory failure, acute infection, bed rest (>3 days), lower limb edema, adenocarcinoma, T-stage, N-stage, M-stage, clinical stage, D-dimer level >1.47 μg/mL, CEA >10.935 ng/mL, and COMPASS-CAT score were related to an increased VTE risk. However, transfusion, previous history of VTE, WBC >8.605 × 10^9^/L, Fbg >4.895 g/L, NSE >12.845 μg/L, and CY211 > 9.765 μg/L were only related to an increased VTE risk in the primary cohort (*P=*<0.05). Chemotherapy was only related to an increased VTE risk in the validation cohort (*P=*<0.05) (Table 3).

**TABLE 3:** Univariate and multivariate analyses for VTE risk factors in lung cancer patients.

Variable		Univariate analysis			Multivariate analysis		
		HR	[95% CI]	*P* value	HR	[95% CI]	*P* value
Primary cohort							
Sex	Male (vs. female)	0.983	[0.621–1.555]	0.940			
Age	>60 (vs. ≤60)	1.367	[0.894–2.089]	0.149			
Smoking status	Yes (vs. No)	1.051	[0.688–1.605]	0.818			
BMI (kg/m^2^)	≥25 (vs. <25)	0.527	[0.238–1.168]	0.115			
Hypertension	Yes (vs. No)	0.979	[0.605–1.585]	0.932			
Diabetes	Yes (vs. No)	1.397	[0.763–2.559]	0.278			
Coronary heart disease	Yes (vs. No)	2.037	[0.917–4.525]	0.081			
Respiratory failure	Yes (vs. No)	4.650	[2.265–9.549]	<0.001			
Acute infection	Yes (vs. No)	5.973	[3.657–9.756]	<0.001	3.056	[1.472–6.342]	0.003
Bed rest (>3 days)	Yes (vs. No)	4.109	[2.643–6.388]	<0.001	1.928	[1.025–3.626]	0.042
Lower limb edema	Yes (vs. No)	4.348	[1.875–10.082]	0.001			
Transfusion	Yes (vs. No)	4.406	[1.221–15.898]	0.024			
Central venous catheter	Yes (vs. No)	1.981	[0.924–4.248]	0.079			
VTE history	Yes (vs. No)	8.456	[2.638–27.107]	<0.001			
Pathology	Adenomatous carcinoma (vs. Others)	2.354	[1.427–3.881]	0.001	2.059	[1.024–4.136]	0.043
Differentiation	Poorly (vs. High/Moderately)	1.347	[0.367–4.945]	0.653			
T-stage	/	1.640	[1.351–1.991]	<0.001			
N-stage	/	1.750	[1.394–2.197]	<0.001			
M-stage	/	2.061	[1.305–3.252]	0.002			
Clinical stage	/	1.896	[1.382–2.602]	<0.001			
Gene mutation	Mutant (vs. wild)	0.688	[0.350–1.352]	0.278			
EGFR+	Mutant (vs. wild)	0.800	[0.415–1.542]	0.505			
ALK+	Mutant (vs. wild)	0.816	[0.272–2.449]	0.717			
D-dimer level >1.47 μg/mL	Yes (vs. No)	5.658	[3.393–9.435]	<0.001	3.471	[1.812–6.649]	<0.001
White blood cell count >8.605 × 10^9^/L	Yes (vs. No)	3.534	[2.281–5.473]	<0.001			
Platelet count >349.5 × 10^9^/L	Yes (vs. No)	1.632	[0.916–2.909]	0.097			
Fbg >4.895 g/L	Yes (vs. No)	1.883	[1.172–3.025]	0.009			
CEA >10.935 ng/mL	Yes (vs. No)	2.579	[1.659–4.009]	<0.001	1.912	[1.026–3.563]	0.041
NSE >12.845 μg/L	Yes (vs. No)	2.444	[1.559–3.831]	<0.001			
CY211 > 9.765 μg/L	Yes (vs. No)	2.336	[1.522–3.585]	<0.001			
proGRP >38.165 ρg/mL	Yes (vs. No)	1.515	[0.870–2.636]	0.142			
SCC >0.33 ng/mL	Yes (vs. No)	3.120	[0.710–13.714]	0.132			
Surgery	Yes (vs. No)	0.624	[0.303–1.286]	0.202			
Only chemotherapy	Yes (vs. combination therapy)	1.938	[0.980–0.382]	0.057			
Targeted therapy	Yes (vs. No)	0.858	[0.443–1.661]	0.649			
Antiangiogenic therapy	Yes (vs. No)	1.151	[0.523–2.535]	0.727			
Immunotherapy	Yes (vs. No)	2.933	[0.758–11.349]	0.119			
Radiotherapy	Yes (vs. No)	1.692	[0.790–3.626]	0.176			
COMPASS-CAT	/	1.675	[1.498–1.872]	<0.001	1.608	[1.411–1.833]	<0.001
Validation cohort							
Sex	Male (vs. female)	1.825	[0.964–3.456]	0.065			
Age	>60 (vs. ≤60)	1.074	[0.575–2.007]	0.822			
Smoking status	Yes (vs. No)	0.897	[0.481–1.676]	0.734			
BMI (kg/m^2^)	≥25 (vs. <25)	0.290	[0.065–1.294]	0.105			
Hypertension	Yes (vs. No)	1.531	[0.769–3.050]	0.225			
Diabetes	Yes (vs. No)	1.231	[0.458–3.307]	0.680			
Coronary heart disease	Yes (vs. No)	0.617	[0.173–2.208]	0.458			
Acute myocardial infarction	Yes (vs. No)	3.510	[0.482–25.564]	0.215			
Respiratory failure	Yes (vs. No)	2.917	[1.029–8.270]	0.044			
Acute infection	Yes (vs. No)	5.616	[2.861–11.024]	<0.001	3.948	[1.549–10.063]	0.004
Bed rest (>3days)	Yes (vs. No)	3.106	[1.633–5.907]	0.001			
Lower limb edema	Yes (vs. No)	5.377	[1.628–17.757]	0.006			
Transfusion	Yes (vs. No)	0.847	[0.174–4.120]	0.837			
Central venous catheter	Yes (vs. No)	1.800	[0.640–5.063]	0.265			
VTE history	Yes (vs. No)	2.656	[0.575–12.277]	0.211			
Pathology	Adenomatous carcinoma (vs. Others)	10.769	[3.227–35.939]	<0.001	10.936	[2.501–47.831]	0.001
T-stage	/	1.671	[1.235–2.261]	0.001			
N-stage	/	1.650	[1.189–2.289]	0.003			
M-stage	/	2.214	[1.102–4.449]	0.026			
Clinical stage	/	1.581	[1.028–2.433]	0.037			
Gene mutation	Mutant (vs. wild)	1.425	[0.346–5.873]	0.624			
EGFR+	Mutant (vs. wild)	0.894	[0.253–3.157]	0.862			
ALK+	Mutant (vs. wild)	5.700	[0.718–45.260]	0.100			
D-dimer level >1.47 μg/mL	Yes (vs. No)	8.991	[3.410–23.708]	<0.001	7.281	[2.141–24.758]	0.001
White blood cell count >8.605 × 10^9^/L	Yes (vs. No)	1.353	[0.698–2.621]	0.371			
Platelet count >349.5 × 10^9^/L	Yes (vs. No)	1.355	[0.561–3.274]	0.499			
Fbg >4.895 g/L	Yes (vs. No)	0.873	[0.400–1.903]	0.732			
CEA >10.935 ng/mL	Yes (vs. No)	2.080	[1.102–3.927]	0.024			
NSE >12.845 μg/L	Yes (vs. No)	1.736	[0.895–3.369]	0.103			
CY211 > 9.765 μg/L	Yes (vs. No)	1.370	[0.726–2.586]	0.331			
proGRP >38.165 ρg/mL	Yes (vs. No)	1.134	[0.546–2.354]	0.736			
SCC >0.33 ng/mL	Yes (vs. No)	0.376	[0.081–1.740]	0.211			
Surgery	Yes (vs. No)	0.393	[0.132–1.170]	0.093			
Only chemotherapy	Yes (vs. combination therapy)	8.878	[1.940–40.637]	0.005			
Targeted therapy	Yes (vs. No)	0.243	[0.056–1.064]	0.061			
Antiangiogenic therapy	Yes (vs. No)	1.031	[0.265–4.011]	0.965			
Immunotherapy	Yes (vs. No)	1.905	[0.100–8.201]	0.929			
Radiotherapy	Yes (vs. No)	0.531	[0.142–1.985]	0.347			
COMPASS-CAT	/	1.411	[1.239–1.606]	<0.001	1.480	[1.253–1.747]	<0.001

VTE, venous thromboembolism; BMI: body mass index; EGFR, epidermal growth factor receptor; ALK, anaplastic lymphoma kinase; ROS-1, ROS proto-oncogene 1; Fbg, fibrinogen; CEA, carcinoembryonic antigen; NSE, neuron-specific enolase; CY21-1, cytokeratin 19 fragment; ProGRP, gastrin-releasing peptide precursor; SCC, squamous cell carcinoma antigen.

The multivariate analysis further indicated that acute infection, adenocarcinoma, D-dimer level >1.47 μg/mL, and COMPASS-CAT score were independent predictors of VTE risk in LC patients from both cohorts. Additionally, the multivariate analysis identified that bed rest (>3 days) and CEA >10.935 ng/mL were independent predictors of VTE risk, but only for LC patients in the primary cohort (Table 3).

### 3.4 Nomogram construction to predict VTE risk for LC patients

**FIGURE 2:**
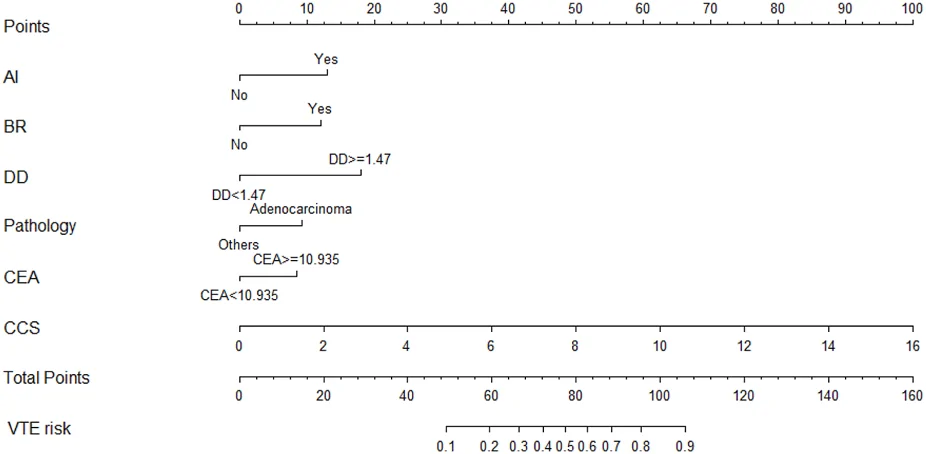
Nomograms to predict VTE risk for lung cancer patients. AI, Acute infection; BR, Bed rest (>3 days); DD, D-dimer level; CEA, carcinoembryonic antigen; CCS, COMPASS-CAT score; VTE, venous thromboembolism.

The area under the AUC curve was greatest for the COMPASS-CAT score model, which these results demonstrated had the best diagnostic value. Based on the multivariate analysis, the COMPASS-CAT score was expanded to establish a nomogram VTE prediction model that is tailored to LC patients, as shown in Figure 2. The total score was the sum of six risk factors: acute infection, bed rest (>3 days), D-dimer level >1.47 μg/mL, adenocarcinoma, CEA >10.935 ng/mL, and the COMPASS-CAT score. The parameters for each factor in these variables were assigned a score within the model to facilitate a cumulative total risk score (Figure 2).

The COMPASS-CAT risk assessment model consists of eight risk factors: anthracycline or anti-hormonal therapy, time since cancer diagnosis, central venous catheter placement, advanced malignancy, history of venous thromboembolism, cardiovascular risk factors (history of peripheral arterial disease, hypertension, hyperlipidemia, diabetes, obesity, coronary artery disease, or ischemic stroke), recent hospitalization for acute illness, and platelet count. In contrast, the variables included in our predictive model are acute infection, bed rest (>3 days), D-dimer level >1.47 μg/mL, adenocarcinoma, and CEA >10.935 ng/mL. None of the variables in our model overlap with the components of the COMPASS-CAT score. Multicollinearity diagnosis was also performed, and the variance inflation factor (VIF) was <10, confirming a low likelihood of multicollinearity.

### 3.5 Nomogram evaluation and verification

**FIGURE 3:**
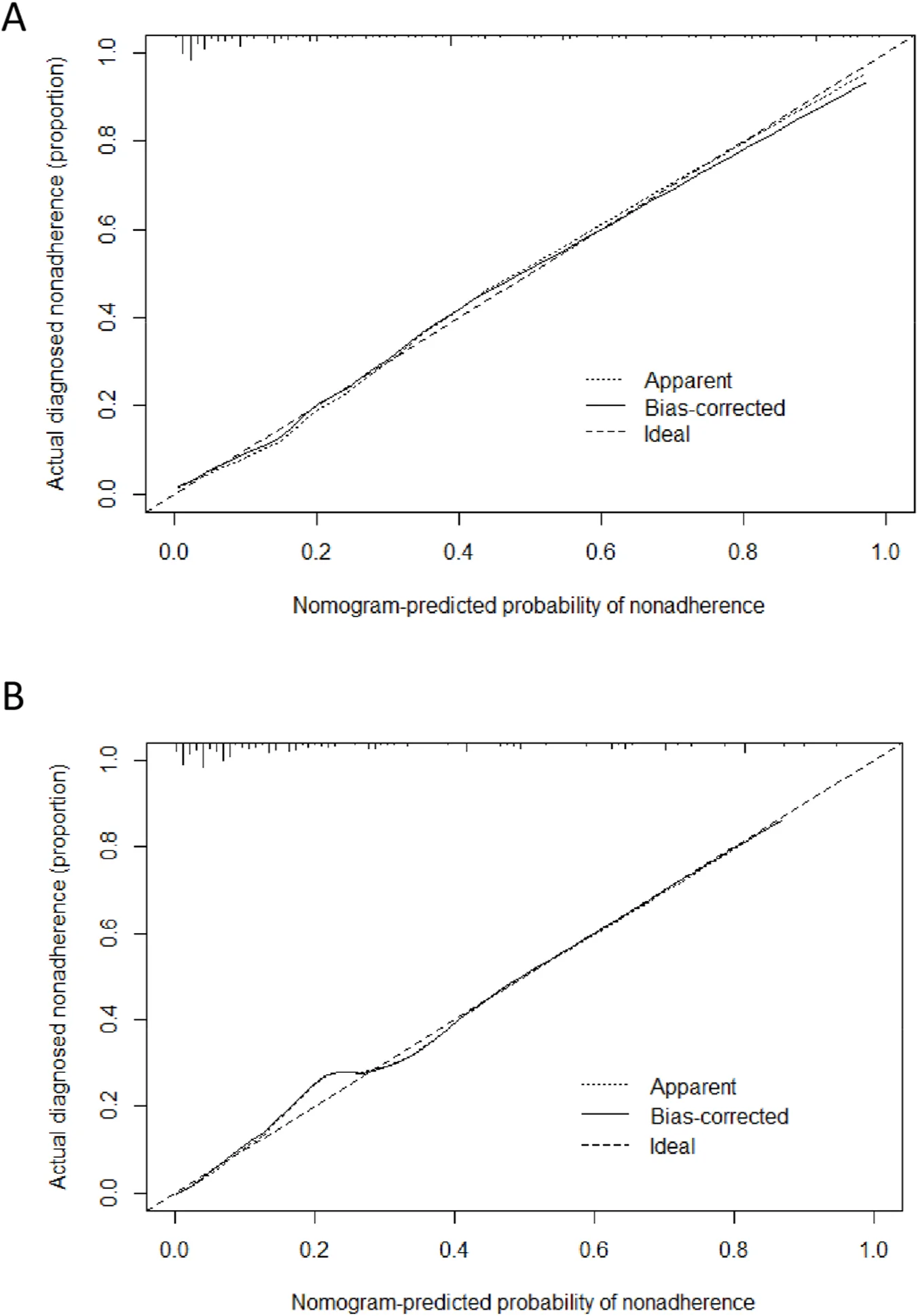
Nomogram model calibration curves for the primary **(A)** and validation cohorts **(B)**. The 45° reference line indicates the expected trend for perfect calibration.

The C-index for this VTE prediction nomogram was 0.893 (0.860–0.926) in the primary cohort, and 0.882 (0.830–0.934) when derived for the validation cohort. Hence, the nomogram demonstrated good predictive accuracy for LC-associated VTE risk in both cohorts. A calibration curve was also drawn to investigate the consistency between the predicted and real values, which showed that the nomogram closely corresponded with the 45° ideal curve lines in both the primary and validation cohorts (Figure 3). This analysis indicated that the nomogram demonstrated agreement between the forecasted probabilities and actual observations.

**FIGURE 4:**
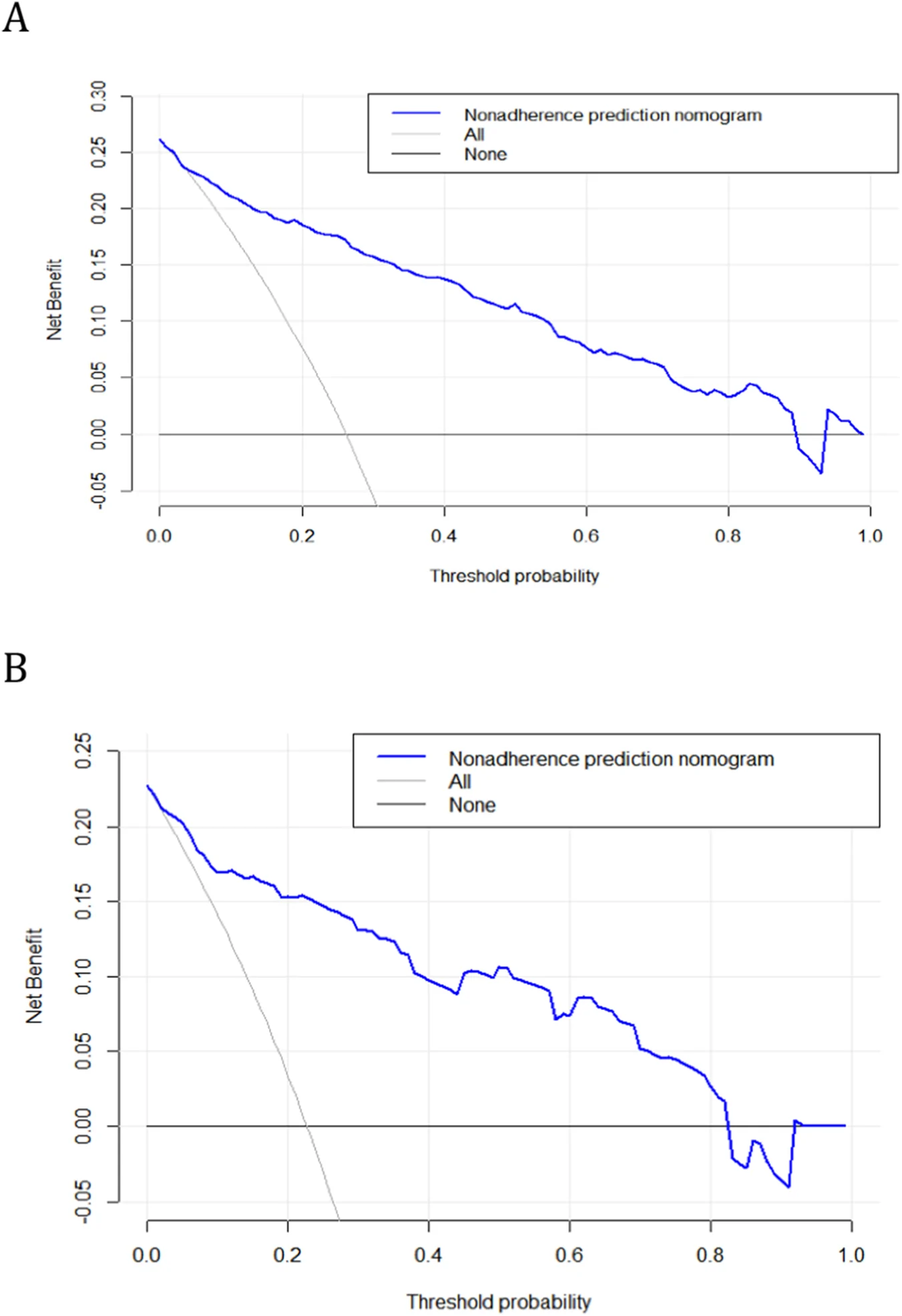
Nomogram model decision curve analysis for the primary **(A)** and validation cohorts **(B)**. The blue line represents the net clinical benefit relative to the threshold probabilities. The horizontal line represents the trend for patients who did not receive an intervention; hence, the net gain was 0. The diagonal line represents the expected trend if all the patients had received the intervention.

The DCA curve is a widely used clinical utility that can be used to analyze the net clinical benefit for a nomogram. The distance between the net benefit line and intervention thresholds within the DCA curve indicated that the nomogram has good clinical practicability and accurate VTE risk prediction in LC patients (Figure 4).

In addition, after excluding the COMPASS-CAT score in the sensitivity analysis, the C-index was 0.809 (95% CI: 0.773–0.846) in the primary cohort and 0.828 (95% CI: 0.754–0.858) in the validation cohort. Although the overall predictive performance in both cohorts was slightly lower than that of the original full model (0.893, 95% CI: 0.860–0.926 in the primary cohort and 0.882, 95% CI: 0.830–0.934 in the validation cohort), the C-index values remained above 0.8, indicating favorable and robust discriminative ability.

**FIGURE 5:**
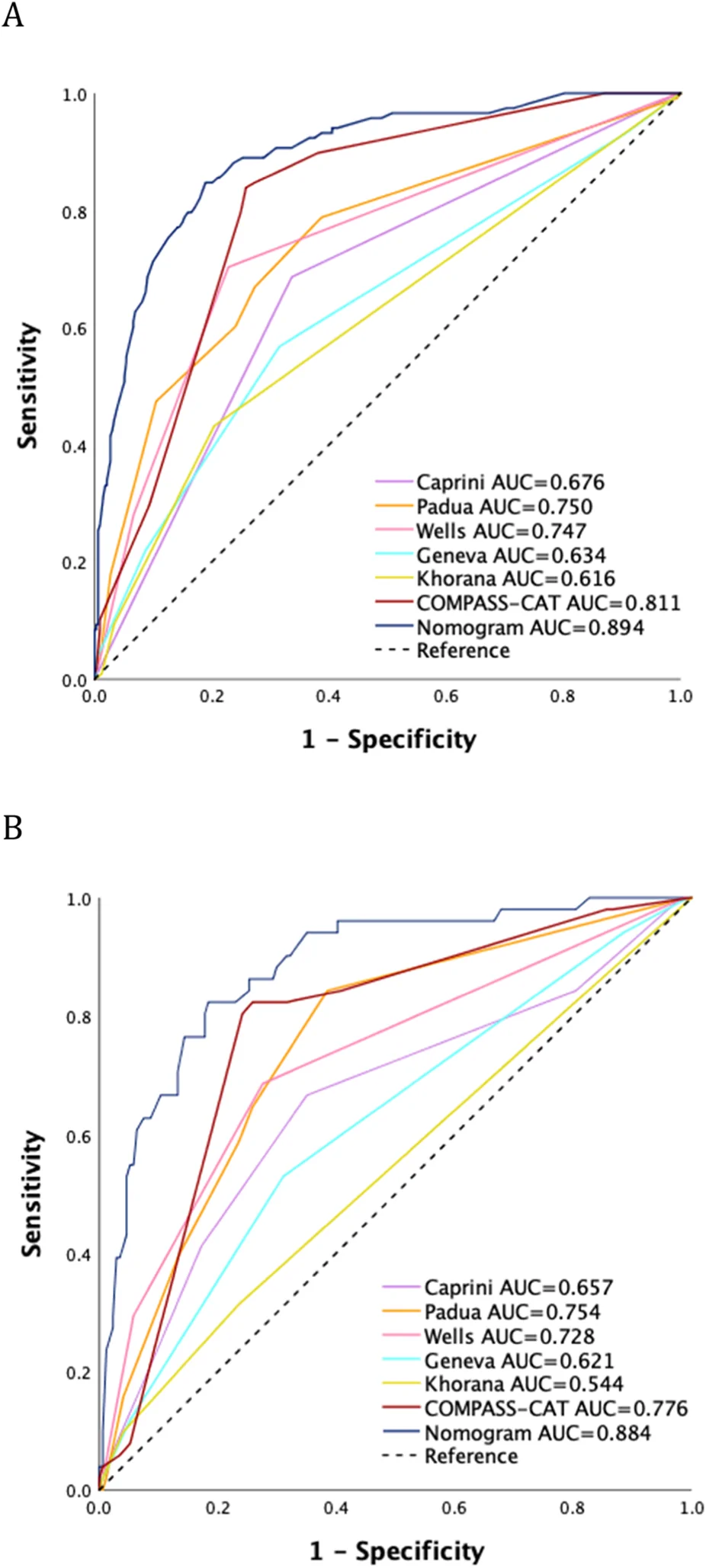
Nomogram predictive performance compared with Khorana, Caprini, Padua, COMPASS-CAT, Wells, and Geneva models. Receiver operating characteristic (ROC) curves showing VTE risk for lung cancer patients in the primary **(A)** and validation cohorts **(B)**.

The predictive value of the nomogram and the six VTE risk scores was evaluated by comparing the AUCs. The AUCs of the nomogram, Caprini, Padua, Wells, Geneva, Khorana, and COMPASS-CAT scores were 0.894, 0.676, 0.750, 0.747, 0.634, 0.616, and 0.811 (Figure 5A), respectively. These values indicated that the nomogram was superior to the other risk scores. Similar results were confirmed in the validation cohort (Figure 5B). Given the small number of patients in the validation cohort, we performed 5-fold cross-validation. The final results showed that the mean AUC was 0.869 ± 0.075, and the mean accuracy was 0.849 ± 0.051. Additionally, the predictive value exhibited a stable positive correlation with VTE risk. This model can serve as an effective tool for VTE risk assessment and is worthy of further clinical validation and application.

## 4 Discussion

In our study, we found that the COMPASS-CAT score was significantly more effective than the other scores when identifying LC patients at high risk of VTE. The AUC for the COMPASS-CAT score was 0.799, but its overall predictive performance was still low. The consistency between the predicted risk by the COMPASS-CAT model and the actual VTE risk was poor. In addition, 90.4% of LC patients can be assigned to a high-risk group according to a retrospective study that explored the use of COMPASS-CAT for 1108 LC patients (Spyropoulos et al., 2020). Rupa-Matysek et al. (2018) also reached similar conclusions. They found that 67.8% of LC patients are placed in the high-risk group, which means many patients are classified as high risk and do not develop VTE. COMPASS-CAT was established using patients with various types of cancer, as opposed to being LC patient-specific. The results from both the aforementioned studies and presented herein reinforce the notion that a reliable LC-specific model for VTE prediction is needed.

There is broad agreement in the medical literature regarding the risk factors associated with VTE events in LC patients. These consist of cancer type, treatment, biomarker, and patient-related factors. The adenocarcinoma histological type, clinical stage, and time interval after diagnosis are well-recognized LC-associated VTE risk factors (Vitale et al., 2015; Dimakakos et al., 2018; Di et al., 2021; Yan et al., 2021). However, the association of oncogene mutations with VTE remains controversial for LC patients. A systematic review of 20 retrospective studies showed that ALK mutations were associated with a higher VTE risk than EGFR mutations (Liu et al., 2021), while another systematic review reported that the EGFR mutation was the strongest risk factor for LC-associated VTE (Alexander and Burbury, 2016). Qian et al. (2021) conducted a systematic review of 25 studies that found no significant relationship between VTE risk and EGFR mutation in LC patients. They also found that an ALK mutation occurred in 29.6% of the patients with VTE, which is higher than the rate found in general non-small cell lung cancer (NSCLC) patients (ALK: 5%). However, the higher ALK mutation prevalence could be due to an association with longer survival times when compared to other driver genes, which could account for the higher VTE incidence observed in these patients. Other studies performed survival analyses to prove that the increased risk of VTE was not due to prolonged survival (Ng et al., 2019; Dou et al., 2020). These conflicting results may explain the lack of significance of genetic mutations in our study.

The conventional treatment for LC patients includes surgery, chemotherapy, and targeted therapy, of which surgery has long been recognized as a risk factor for VTE (Li and Jiang, 2018). However, only the univariate analysis (included herein) highlighted that surgery was an independent prognostic factor for VTE. Kadlec et al. (2014) found that LC patients who underwent surgery had a 17.6% incidence of VTE. However, the use of pharmaceutical anticoagulants, mechanical measures, and early ambulation has led to a dramatic reduction in the incidence (to 0.18%) of postoperative VTE (Gómez-Hernández et al., 2013). The use of these measures may account for the reduced influence of surgery on VTE incidence in our cohort and lack of significance in the multivariate analysis.

Chemotherapy drugs commonly used to treat lung cancer (e.g., platinum-based chemotherapies and gemcitabine) have been found to increase the risk of VTE (Moore et al., 2011; Rupa-Matysek et al., 2018). In our study, the association between chemotherapy and VTE was significant in the univariate analysis but was not significant in the multivariate analysis. The mechanisms by which chemotherapy contributes to VTE development have not yet been fully elucidated. Nevertheless, the downregulation of endogenous anticoagulants and vascular endothelial damage (especially in patients receiving medication through a central venous catheter over a long period) are thought to be important contributors (Hohl Moinat et al., 2014; Kuderer et al., 2018; Di et al., 2021). A recent study of 1587 patients found that a higher VTE risk was identified in patients who received treatments other than chemotherapy alone (compared to chemotherapy alone). The 6-month cumulative VTE incidence by treatment type was: 5.0% (chemotherapy), 7.6% (immune checkpoint inhibitors; ICIs), 9.9% (chemotherapy plus concomitant ICIs), 9.4% (chemotherapy and durvalumab), and 11.1% (targeted therapies; TTs). The 12-month incidence was 6.5% (chemotherapy), 9.0% (ICIs), 12.8% (chemotherapy plus concomitant ICIs), 12.2% (chemotherapy and durvalumab), and 13.1% (TTs) (Hill et al., 2021). Therefore, it appears that many treatments can affect VTE, and the clinical situation is becoming more complicated. Fewer patients received chemotherapy alone, and further work is needed to determine the impact of combination therapies on VTE.

The role of radiation therapy, TTs, and ICIs in VTE development has been evaluated in some studies with conflicting results (Blom et al., 2004; Blom et al., 2006; Lin et al., 2006; Hurwitz et al., 2011; Corrales-Rodriguez et al., 2014; Salla et al., 2016; Sato et al., 2019; Giustozzi et al., 2021; Hill et al., 2021; Moik et al., 2021; Allouchery et al., 2022). Our study showed that radiation therapy, TTs, and ICIs were not associated with VTE risk. However, Hill et al. (2021) found that TTs were associated with a higher risk of VTE than other treatments. A different study found that patients with lung adenocarcinoma were at a reduced VTE risk when treated with TTs (compared to other treatment strategies not including TTs) (Corrales-Rodriguez et al., 2014). Some studies found that gefitinib and erlotinib did not appear to increase the risk of VTE events (Salla et al., 2016), whilst other studies suggest the opposite conclusion (Hurwitz et al., 2011). ICI treatment in cancer patients has also been associated with high VTE risk (Giustozzi et al., 2021; Moik et al., 2021), due to the possible induction of procoagulant effects (Sato et al., 2019). However, Ma et al. (2022) found there was no significant overall increased VTE risk in patients treated with ICIs in a meta-analysis that included 61 studies. An analysis by Allouchery et al. (2022) using the World Health Organization pharmacovigilance database provided a similar conclusion. Given the conflicting results derived by previous studies, it remains prudent to exclude TTs and ICIs from our nomogram for LC patients. However, because TTs and ICIs have become pillars of LC treatment, further prospective studies are needed to assess the impact of these therapies on VTE risk.

Platelet counts have also been identified as a VTE biomarker and are incorporated in many VTE risk scores (Khorana et al., 2008; Gerotziafas et al., 2017). D-dimer level is a well-known biomarker for thrombosis that reflects fibrinolytic activity. Biomarkers such as p-selectin, fibrinogen, activated partial thromboplastin time, and other coagulation-related indicators have also been associated with VTE occurrence in LC patients but lack external validation for their predictive roles (Vitale et al., 2015; Di et al., 2021; Yan et al., 2021). Additionally, inflammatory mediators (particularly cytokines that are associated with vascular inflammation) have recently been proposed as predictors for use in new VTE prediction models but warrant further evaluation (Vitale et al., 2015; Di et al., 2021; Yan et al., 2021).

Unlike other VTE risk scores that were established using patients with various cancer types, our nomogram utilized data for only LC patients and thereby contains information that is specific to LC patients, such as histological subtype (adenocarcinoma) and CEA >10.935 ng/mL. The nomogram presented herein facilitated a good predictive ability (AUC = 0.894 and C-index = 0.893) based upon data from 676 LC patients. The calibration curve showed that the predicted probability for the model was in high agreement with the actual predicted probability, and the DCA curve showed that the model had great potential clinical utility. Although our nomogram showed good predictive ability for LC patients, a prospective cohort is required for validation.

In recent years, artificial intelligence/machine learning (AI/ML) models have become powerful tools for clinical risk prediction (Al Raizah and Alrizah, 2025; Ma et al., 2025), among which there are also VTE prediction models constructed specifically for lung cancer patients (Lei et al., 2022; Chen et al., 2025). Unlike traditional scoring systems that rely on pre-defined clinical variables and fixed weights, AI/ML models have unique advantages: they can automatically capture the complex non-linear relationships between predictors and VTE outcomes and simultaneously identify potential risk factors that are easily overlooked by traditional statistical methods, thereby further improving the accuracy of risk prediction. However, AI/ML also has limitations. Some complex AI/ML models have a “black-box” problem with poor interpretability, which does not help clinicians understand the underlying mechanisms of prediction results, thereby affecting clinical acceptance (Al Raizah and Alrizah, 2025). A recent study on the application of machine learning in VTE prediction also pointed out that most of the included studies (18/27, 67%) were found to have a high risk of bias, mainly due to insufficient handling of missing data and inadequate reporting of study design (Ma et al., 2025). Therefore, the clinical accuracy of AI/ML models must be further verified, and there are certain obstacles to their popularization and application in clinical practice.

In contrast to AI/ML models, the nomogram model in our study is more intuitive, balancing interpretability and clinical practicality, and is more in line with the current routine clinical application scenarios. Of course, we recognize the application value of AI/ML models in VTE risk prediction. In the future, we will expand the sample size, integrate multi-center and multi-dimensional data, and use AI/ML technology to optimize our nomogram model and improve the efficiency of risk stratification, so as to provide more accurate individualized VTE prevention strategies for lung cancer patients.

Second, the prediction model developed in this study still has limitations; for instance, it cannot fully characterize the complex biological mechanisms underlying disease susceptibility. As emphasized by Ruan and Yi (2025), future risk prediction will focus on unraveling shared genetic architecture between comorbidities, a gap we aim to address via genetic-level analyses to deepen the model. Additionally, we acknowledge the growing emphasis on advanced visualization methods to enhance real-world applicability in contemporary high-quality oncology research. As highlighted by Rong et al. (2025), web-based dynamic predictive tools offer distinct advantages over traditional static nomograms, including intuitive user interfaces, real-time risk calculation, and seamless integration with clinical workflows, which are features that significantly improve adoption among frontline clinicians and optimize decision-making efficiency. As the robustness of the model is validated, it can also be integrated with hospital electronic health record (EHR) systems to automatically extract key variables from patient medical records, thereby generating real-time VTE risk stratification results. Through a user-friendly interface, patients’ risk probabilities are clearly displayed, and high-risk cases are marked with visual alerts, assisting clinicians in promptly formulating diagnosis and treatment decisions and strengthening patient condition monitoring. With the development of LC research and advances in treating LC, we will update this nomogram in order to maintain prediction performance.

## 5 Conclusion

This study identified independent risk variables for venous thromboembolism in lung cancer patients and established a clinical prediction model. The model demonstrated favorable discriminative ability and good calibration in the studied cohorts, and its predictive performance appeared to be superior to several widely used risk assessment tools. Ultimately, this easy-to-use nomogram may serve as a preliminary reference to assist clinical decision making for thromboprophylaxis in high-risk populations. Further prospective validation is warranted before widespread clinical application.

## Data Availability

The raw data supporting the conclusions of this article will be made available by the authors, without undue reservation.
